# Why Do Radiologists Disown Breast Thermography? A Critical Review of Recent Studies and Recommendations

**DOI:** 10.3390/cancers17132195

**Published:** 2025-06-29

**Authors:** Ane Goñi-Arana, Jorge Pérez-Martín, Francisco Javier Díez

**Affiliations:** Department of Artificial Intelligence, Universidad Nacional de Educación a Distancia (UNED), 28040 Madrid, Spain; jperezmartin@dia.uned.es (J.P.-M.); fjdiez@dia.uned.es (F.J.D.)

**Keywords:** breast cancer, thermography, infrared imaging, screening

## Abstract

Thermography was first applied to breast cancer detection in the 1950s but fell out of favor among radiologists due to inconsistent and inconclusive findings in the following decades. Studies conducted in the 21st century using new-generation thermal cameras and computer vision techniques, particularly artificial intelligence, have reported sensitivity and specificity values comparable to those of mammography. However, most radiologists, being unaware of these results, still believe this technique is ineffective, and medical societies advise against using it, even as an adjunct to mammography. In this paper we review recent studies and discuss whether the recommendations of scientific societies are still valid in the light of new evidence. We also propose some ideas for standardizing breast thermography studies that could help make this technique acceptable to the radiology community.

## Simple Summary

Early diagnosis of breast cancer is key to a successful treatment. Thermography is a safe and painless imaging technique with potential as a complementary tool for breast cancer screening and diagnosis. Although recent studies have reported promising results, health agencies oppose its implementation in clinical practice. In this work, we review the evidence supporting breast thermography and critically assess the arguments used by various health authorities to recommend against its use.

## 1. Introduction

Breast cancer caused around 700,000 deaths in 2022 [1]. Early detection is essential to increasing the survival rate and reducing economic costs. In high-income countries, screening mainly relies on mammography, which is sometimes supplemented by ultrasound and magnetic resonance imaging (MRI) [2]. Mammography is very sensitive and specific in general, but it requires expensive equipment and highly trained radiologists to interpret the images, may induce the cancer it intends to prevent, and causes patients’ discomfort, which reduces adherence to screening. Additionally, its sensitivity decreases with breast density, which is in turn a risk factor for breast cancer [3]. Ultrasonography is less costly and radiation-free, but it has lower sensitivity. MRI is highly sensitive but even more expensive than mammography; it takes more time to acquire and interpret the images and sometimes requires intravenous contrast agents to enhance lesion detection.

**Figure 1:**
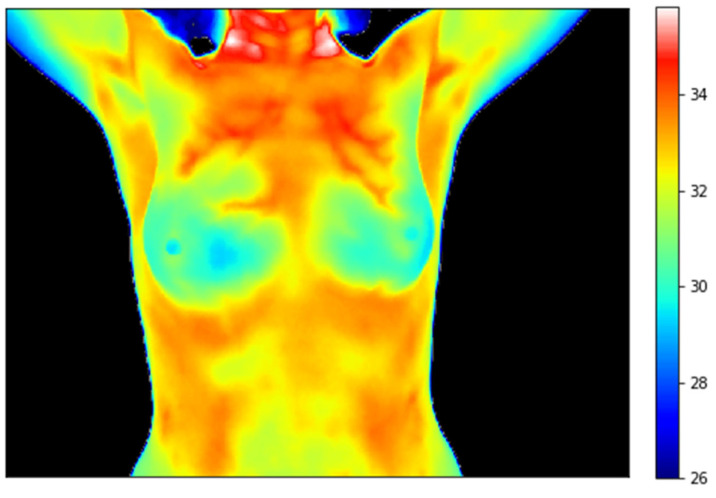
Breast thermogram.

Clinical thermography measures skin temperature by recording the infrared radiation emitted naturally by the human body. Figure 1 shows an example of a breast thermogram. The fact that malignant tumors generate more heat than healthy tissues, due to increased angiogenesis, can be used to detect breast cancer. Thermography may be especially useful in populations for whom mammography is less sensitive, such as women with dense breasts. Since it does not use ionizing radiation, screening could begin at a younger age. Additionally, in low-income countries, where mammography screening is unaffordable due to high costs and a shortage of trained personnel, a combination of ultrasound and thermography can be a viable alternative.

Some researchers have advocated another use of thermography, taking into account that 80% of the biopsies performed because of a positive mammogram turn out to be negative. In the case of a relatively small suspicion of cancer, such as BI-RADS 4A, thermography could be useful because a negative result might reduce the probability of malignancy to the point of making a biopsy unnecessary.

Although recent data show the potential of breast thermography, national health agencies and scientific organizations refuse to include it in their screening programs because of the mixed results reported in the past. The purpose of this paper is to critically review the recent literature on this topic and to evaluate the validity of the arguments—in particular, the clinical studies—on which these institutions base their positions. In other words, we aim to discuss whether the negative recommendations against thermography still hold in light of recent research. Additionally, since the heterogeneity and poor quality of many studies are causes of this rejection, we propose ideas to standardize breast thermography research, in the hope that this will help convince the radiology community of its effectiveness.

Note that there is an alternative thermographic technique that consists of placing on the skin liquid-crystal films that change color depending on the temperature, but we do not discuss it in this paper. Instead, we focus exclusively on infrared thermography.

## 2. Clinical Evidence

### 2.1. Rise and Fall of Thermography in the 20th Century

Thermography began to be studied as a diagnostic tool for breast cancer in the 1950s after surgeon Ray N. Lawson discovered that the skin overlaying a tumor is hotter than in healthy areas [4]. Dozens of scientific articles were published in the following years, but the results reported varied widely, with sensitivity ranging between 25% and 100%, and specificity between 22% and 96% [5,6,7,8]. While some authors claimed that thermography should play an important role in screening or diagnosis of breast cancer [9,10,11,12,13,14,15,16,17,18,19,20], others argued that it does not add any value to the existing screening strategies [21,22,23,24,25]. Moskowitz et al. even concluded that thermography was not able to detect cancer at a rate greater than chance [26]. As a consequence of such contradictory results and the promising results obtained in 1976 by the first large-scale randomized controlled trial of mammographic screening [27], interest shifted to mammography exclusively.

The lasting skepticism towards breast thermography is in great measure due to the Breast Cancer Detection and Demonstration Project (BCDDP), a large-scale multi-center study conducted between 1973 and 1981 and sponsored by the American Cancer Society and the National Cancer Institute (NCI). Its aim was to evaluate the feasibility of periodically screening large numbers of women for breast cancer [28]. Over 280,000 volunteers were screened annually for five years with clinical examination, mammography, and thermography, with an additional five-year follow-up. The low sensitivity obtained for thermography in the first two annual screenings—37% and 44%, respectively—led to the decision to drop this technique from the trial [29,30].

However, the validity of this study has been questioned owing to the negligent methodology used for infrared imaging. According to the physicians that led two of the sites involved, “only five of the 27 BCDDP centers had personnel experienced in either obtaining or reading thermograms”, and no training was provided during the first 18 months [8]. Consequently, in most of the other 22 centers, “thermograms obtained were of terrible quality” [29]. A retrospective study found that only 29% of a subset of 576 randomly selected thermograms from the BCDDP were of acceptable quality [31]. As a result, the “wonderful statistics” from the five centers that had previous experience with thermography were diluted when combined with the rest of the centers [29]. Moreover, the acquisition protocol and the interpretation criteria for infrared imaging were very loosely defined, in just one paragraph, in contrast with the thorough protocol for mammography. Many of the project sites were mobile imaging vans with poor heating and cooling capabilities. It was not until three years after the project began that the NCI published the criteria for reading breast thermograms based on the number, caliber, location and configuration of blood vessels, thermal asymmetry, and breast contour [32].

### 2.2. Resurgence of Breast Thermography in the 21st Century

**Figure 2:**
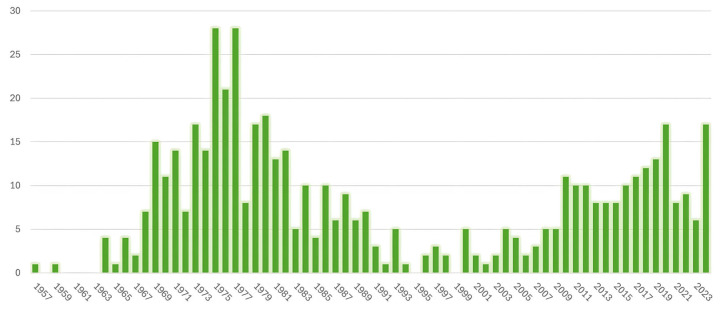
Number of publications about breast thermography in PubMed, on 13 January 2025. Search term: “breast” AND (“thermography” OR “thermographic” OR “thermal imaging” OR “thermal infrared” OR “thermogram” OR “infrared imaging” OR “infrared thermography”) in the title.

With improved silicon technology, smaller and faster infrared detectors were developed in the late 1990s, resulting in new digital thermal cameras with higher thermal and spatial resolution. The time required to obtain a thermogram was reduced from several minutes to milliseconds [33,34]. Involuntary chest motion and skin temperature oscillations during the acquisition no longer lead to blurry images. In addition, digitalization enabled more objective quantifiable interpretation criteria. A wide range of features can be computed from pixel intensity and then compared between the two breasts to look for asymmetries. With the boom of artificial intelligence (AI), numerous image processing algorithms have been proposed to interpret thermograms with minimal or no human interaction [35]. Research has also focused on the automatic detection of areas of increased temperature [36,37] and vasculature [38,39], which can be indicative of malignancy. Such advances led to renewed interest in breast thermography, evidenced by the exponential increase in the number of publications, as shown in Figure 2.

Breast thermography has been the subject of hundreds of scientific articles in the last two decades [40,41,42,43,44]. However, the sample size in most of these studies is small and they focus predominantly on developing novel image processing algorithms rather than clinically evaluating the technology. Consequently, important clinical details such as patient recruitment processes, inclusion and exclusion criteria, imaging protocols, and definitions of positive and negative cases are frequently insufficient or entirely absent. For instance, it is common for authors not to specify whether their patients were attending routine screening (asymptomatic) or being examined due to symptoms or suspicious findings from previous tests. Moreover, ambiguous labels like *healthy* or *normal* and *sick* or *abnormal* are often used without explicitly defining them. Such issues are particularly prevalent in studies using the Database for Mastology Research (DMR), which was the only publicly available dataset until 2024. This database, developed by the Federal Fluminense University and the Federal University of Pernambuco in Brazil, contains thermal images and medical records of 287 patients (239 labeled as healthy, 48 as sick). Although it has been instrumental in facilitating much of the research currently being conducted on automated thermogram classification, many errors and anomalies have been detected in it [45,46], such as identical images assigned to different patients and inconsistent data. Therefore, the results derived from studies relying on this database must be treated with caution.

In contrast, studies evaluating commercial systems in clinical settings are often conducted with a high degree of rigor. However, since these studies are usually supported by the companies that develop and market the imaging system and/or the analysis software used in the study, there is a potential for bias in the results. Several of these studies include the following:

- Parisky et al. [47] tested a system developed by Computerized Thermal Imaging Inc. (Ogden, UT, USA), called BCS 2100, which was intended as an adjunct to mammography to avoid biopsying benign breast masses. These authors examined 769 subjects with 875 lesions biopsied due to abnormal mammographic and/or clinical findings. A series of images were acquired while the patient lay prone with both breasts suspended through openings in the imaging bed and cool air was blown over them. After manually defining the location of the lesions in the mammogram, the proprietary software determined an index of suspicion for the thermogram, which resulted in a sensitivity of 97% and a specificity of 14%. This implies that limiting biopsies to the lesions with a positive thermogram would avoid 14% of unnecessary biopsies of benign lesions, at the expense of missing 3% of the cancers. All the lesions that were assessed as false negative were microcalcifications; excluding these lesions (which were 45% of the total), sensitivity increased to 99% and specificity to 18%. In reply to Moskowitz, who criticized the dependency on mammography to localize the suspicious area in the infrared image, Parisky emphasized that the system was intended to be used in conjunction with mammography to avoid unnecessary biopsies of benign lesions, not to replace it [48]. In 2002, the US Food and Drug Administration (FDA) denied clearance for the BCS 2100 system, and the company became involved in multiple shareholder class-action lawsuits [49].
- Arora et al. [50] assessed the effectiveness of another system, Sentinel BreastScan (SBS), by Infrared Sciences Corp. (Bohemia, NY, USA), in a group of 92 women with suspicious findings on mammography or ultrasound. Among three different analysis modes compared, the one using an artificial neural network obtained the highest sensitivity (96.7%), with a specificity of 26.5%. Although the SBS was cleared by the FDA for adjunctive breast cancer screening in 2004 and obtained the CE Mark, the company seems to have gone out of business.
- In 2010, Wishart et al. [51] evaluated the same SBS system analyzing the images with new software called NoTouch BreastScan (NTBS), by UE Life Sciences Inc. (Philadelphia, PA, USA), which uses AI to detect areas of increased heat. After examining 100 women with 106 lesions biopsied, NTBS obtained a higher sensitivity than the SBS neural network (70% vs. 48%), whereas specificity was lower (48% vs. 74%). In women under 50, the sensitivity of NTBS increased to 78% (the same as mammography) and the specificity to 75%, suggesting that thermography could be particularly helpful for this specific population. In fact, the combination of both techniques raised the sensitivity to 89% for this age group.
- Later, UE Life Sciences Inc. developed a new system, similar to SBS, by incorporating two high-resolution thermal cameras to their NTBS software, each pointing at one breast. Collet et al. [52] independently evaluated it by examining 99 patients prior to scheduled breast biopsy. Depending on the purpose of the examination, NTBS offered two types of analysis: a high specificity *screening mode*, in which the objective is to minimize false positives at the expense of a lower sensitivity, and a high sensitivity *diagnostic mode*, which aims to correctly identify all malignant cases at the cost of a higher false-positive rate. Although the sensitivity (78.8%) and specificity (48.6%) obtained with the diagnostic mode were similar to Wishart’s, Collet et al. did not consider these results good enough to recommend thermography as a screening modality, not even as an adjunct to mammography. In spite of the FDA’s clearance of NTBS as an adjunct to other screening modalities in 2012, UE Life Sciences subsequently switched its strategy to tactile technology for breast cancer detection (their iBreastExam device, https://www.uelifesciences.com/ibreastexam) and the website for NoTouch BreastScan is no longer available.
- A prototype 3D infrared imaging system by Real Imaging Ltd. (Airport City, Israel) was evaluated in 2013 by Sella et al. [53] in 256 healthy women—with BI-RADS-1 results in routine mammography screening—and 178 women with newly diagnosed breast cancer (it is not specified whether they were symptomatic, or cancer was discovered in screening). The device, which has received different names (3DIRI, Real Imager 8, or MIRA), consists of two infrared cameras placed at a 60° angle between them, which produce a 3D thermal map of the breast. AI was used to output a score between −100 (healthy) and 100 (suspicious), with a sensitivity of 90.9% and a specificity of 72.5%. Real Imaging Ltd. sponsored two other studies in which women with a genetic predisposition for breast cancer and women with dense breasts were examined by mammography, ultrasound, and 3DIRI. Patients with negative mammography or ultrasound but an abnormal thermogram were referred to MRI. In the first study [54], 8 cancers were found among the 226 patients at high risk due to genetic predisposition: 7 of them were correctly diagnosed by 3DIRI (87.5% sensitivity), whereas mammography and ultrasound missed 3 of them (60% sensitivity). The specificity for 3DIRI was also high (84.32%). Among 1727 women with dense breasts, thermography detected 6 cancers in 5 women in addition to the 7 cases identified by mammography, thus increasing the detection rate from 4.05 to 6.95 per 1000 [55]. However, 5 of the cancers visible in mammography were missed by 3DIRI. Although the individual sensitivity was low for both mammography and 3DIRI (7 cancers out of 12 detected by each technique, i.e., 58.3%), the combined sensitivity reached 100% (all the cancers were detected), with a specificity of 86% (87% 3DIRI, 98% mammography).
- Thermalytix, a computer-aided diagnostic solution by the start-up Niramai Health Analytix (Bangalore, India), uses AI to compute a risk score based on the presence of asymmetric vascular and thermal patterns (https://www.niramai.com/about/thermalytix). The company funded three studies to evaluate it. Kakileti et al. [56] examined 470 women, 50.6% of which were symptomatic, obtaining a sensitivity of 91.02% (89.85% for symptomatic; 100% for asymptomatic) and a specificity of 82.39% (69.04% for symptomatic; 92.41% for asymptomatic). Singh et al. [57] reported a sensitivity of 82.5% and a specificity of 80.5% after imaging 258 symptomatic women. Bansal et al. [58] used Thermalytix to examine 459 women; 85% were asymptomatic and 36.6% had dense breasts. The overall sensitivity was 95.24% and the specificity 88.58%. Three of the biopsy-proven malignancies had an inconclusive mammography (BI-RADS 0) but an abnormal thermogram. Among the women with dense breasts, which accounted for 57% of women under 45 years, the sensitivity was 100% (the same as mammography) and the specificity 81.65% (superior to that of mammography). Thermalytix obtained CE marking in 2021 and is now commercially available in 22 countries. The following year their SMILE-100 system, which uses a subset of features of Thermalytix, was cleared by the FDA.

We identified a few other studies that we considered noteworthy for including a large number of patients and/or broader population types.

- Gutierrez-Delgado and Vazquez-Luna published the results obtained in a study conducted in Mexico that included 911 women attending cancer screening programs, with physical examination, breast thermography and mammography [59]. Among the 17 biopsy-proven cancers (a higher-than-normal incidence), 16 had an abnormal thermogram (94.12% sensitivity). More importantly, three of the cancers were detected in women under 40 years—a population for which guidelines do not recommend mammography—with a positive thermogram. This detection rate is surprisingly high, considering that images were interpreted with obsolete visual criteria. Unfortunately, the number of false positives was not reported.
- Rassiwala et al. [60] conducted a study, also in a screening setting, in India. Among the 1008 asymptomatic women, 49 had an abnormal thermogram. All of them had a palpable lump in the clinical examination and were subjected to mammography and histopathological examination. In this group, 41 cancers were found, which is a very high incidence; 3 of them were missed by mammography. With only one false-negative, the sensitivity of thermography was 97.62% and the specificity 99.17%. Such good results are unexpected given the low resolution of the camera and the simple interpretation criterion, as the diagnosis was based solely on temperature differences between the breasts.
- Yao et al. [61] examined 2036 women in China who required a biopsy or surgical excision because of abnormal mammographic or ultrasound findings. The sensitivity of thermography (84.4%) was superior to that of mammography (78.3%) and ultrasound (83.1%), whereas specificity (94.0%) was inferior to mammography (98.3%) but superior to ultrasound (93.1%). For tumors smaller than 2 cm in diameter, the sensitivity and specificity of thermography increased to 90.4% and 97.8%, respectively, outperforming mammography (80.8% and 97.6%).
- In Mexico, Garduño-Ramón et al. [62] obtained a sensitivity of 86.84% and a specificity of 89.65% when examining 454 voluntary women; it is not specified how they were recruited. In contrast with the sophisticated algorithms used in other contemporary publications, this study classified thermal images as *healthy* or *sick* by simply comparing the average temperature of the hottest region in each breast.
- Wang et al. proposed using a thermal camera attached to a mobile phone for in-home pre-screening [63]. By applying AI, they obtained an accuracy of 86.27%, sensitivity of 84.51%, and a specificity of 83.87% in 2202 patients recruited from 20 health centers in 10 regions of China.

Table 1 summarizes the studies mentioned in this section.

More recently, Goñi-Arana et al. conducted a systematic review of clinical studies published in the 21st century that assessed the sensitivity and specificity of infrared thermography [64]. Only 22 studies met the established criteria to ensure that the selected studies reported the minimal clinical data necessary for acceptable quality. Their meta-analysis revealed a pooled sensitivity of 88.5% and a specificity of 71.8%. Interestingly, while sensitivity remained consistently high across the studies, specificity increased over time, possibly as a result of improvements in thermal camera resolution. However, most of the selected studies were small and showed significant variability in methodology in terms of patient populations, imaging protocols, camera resolutions, and interpretation methods. Consequently, the *I*^2^ statistic, which estimates the percentage of variability due to differences between studies rather than to sampling errors (chance), returned very high values: 79.3% for sensitivity and 99.1% for specificity. This variability makes direct comparison of the results challenging and highlights the need to standardize methodologies in future research. The review concluded that while breast thermography shows promise, larger studies involving more diverse populations are necessary to establish its role in breast cancer diagnosis.

## 3. Recommendations and Position Statements

The FDA approved thermography as an adjunct to mammography for breast cancer screening in 1982 and cleared a few commercial systems using thermography in the 21st century, as mentioned above. However, this agency has repeatedly shown concern regarding healthcare providers and manufacturers that promote it as a standalone screening technique, saying that the risk of radiation outweighs the benefits of mammography, and even claiming that compressing the breast during mammography may spread cancer cells to other parts of the body. As a response, the FDA has issued warning letters to some of them, insisting that mammography is the only screening technique that has proved to reduce mortality, demanding that they stop making such claims and threatening to take action otherwise [65].

Some medical societies have also issued recommendations against breast thermography. For instance, the Society of Breast Imaging (SBI) stated in 2012 that it “does not currently support the use of thermography/infrared imaging of the breast as either a screening tool in the detection of breast cancer or as an adjunctive diagnostic tool”, supporting this statement with a few studies, which go as far back as 1972. It just cites three studies published in the 21st century [47,50,51], which only include patients with suspicious lesions that were biopsied. So, it presents the BCDDP—conducted in the late 1970s, whose limitations we have discussed above—as the most recent evaluation of breast thermography in a screening setting. It is also noteworthy that the statement takes up the criticisms of Moskowitz et al. about the study by Parisky et al., but does not mention Parisky’s rebuttal, mentioned above, which is in the same document [48]. This statement is no longer available online, but the SBI confirmed its position upon our email inquiry.

Similarly, a “position paper on screening for breast cancer by the European Society of Breast Imaging (EUSOBI) and 30 national breast radiology bodies” published in 2017 states that “these societies strongly discourage the use of methods for screening such as thermography or other optical imaging tools as an alternative to mammography” [66]. They justify their position by referring to a 2013 paper by Brkljačić, Miletić and Sardanelli—then president of EUSOBI—[67], published in a non-medical journal, *Collegium Antropologicum*. This paper was intended as a strong criticism of a small study (29 patients) in the same journal issue, in which thermography was superior to mammography at detecting malignancies in patients undergoing breast carcinoma surgery, leading to the conclusion that thermography might be used in conjunction with mammography [68]. Brkljačić et al. reject that claim by recalling the recommendations of the FDA and the SBI, and offering a brief review of the scientific evidence. They bring up again the poor results obtained in the BCDDP trial [30] and then cite three relatively recent studies that use new-generation thermal cameras with patients who were symptomatic or had suspicious findings in prior imaging tests [50,51,69]. When analyzing the results of Arora et al. [50], they give the mean values for sensitivity and specificity obtained with the three analysis modes, instead of providing the result for the mode with the best sensitivity–specificity balance. Although the sensitivity given (94%) is close to the actual value (96.7%), the implications are important, as the false positive rate (1—sensitivity) is almost twice the real one (6% vs. 3.3%). Brkljačić et al. also cite a study by Kontos et al. [69], in which a sensitivity of only 25% was obtained for 63 symptomatic patients. This study bases the diagnosis on the number of color differences between adjacent areas instead of using actual temperature measurements. In spite of this limitation, the small sample size, and other methodological issues that we cannot discuss here, Brkljačić et al. accept the conclusion of Kontos et al. that “thermography is not indicated for the primary evaluation of symptomatic patients, nor should it be used for breast cancer screening”. In contrast, they do not mention that Arora et al. [50] and Wishart et al. [51] advocate the use of thermography as an adjunctive test for breast cancer detection.

The Department of Health of the Australian Government also advised against the use of thermography for breast cancer screening in 2019, based on the lack of scientific evidence to support it [70]. Their statement only cites two articles, which date back to the 1980s. One of them is used, once again, to recall the poor results of the BCDDP trial [71]. The other is used to argue that thermography can only detect large tumors [72] (despite the fact that Yao et al. had proven otherwise five years earlier [61]). A systematic review commissioned by this institution observed that studies on breast thermography published in the period 2010–2017 used small sample sizes, and that the reported sensitivity and specificity values varied greatly [73]. This review, which focused on breast screening techniques, dismissed several studies with symptomatic patients [51,52,61,62] and concluded that there is a lack of large-scale screening studies and randomized control trials of breast thermography.

## 4. Discussion

As mentioned above, significant technical breakthroughs in digital infrared cameras, as well as the use of computer vision algorithms to analyze images, have led to major advancements in breast thermography. Several clinical studies have reported high sensitivity values with acceptable specificity. These results will continue to improve as AI models are trained using larger datasets. Therefore, breast thermography could be an adjunctive technique for populations in which mammography is less sensitive. Additionally, since it does not use ionizing radiation, screening could begin at a younger age. Thermography can also help distinguish benign from malignant masses detected with other techniques, thus avoiding some biopsies of benign lesions [47,61]. Given its low cost and ease of use, it could have an important impact in low-income countries, where screening with mammography is not possible due to its high cost and shortage of trained personnel.

However, the scientific evidence regarding breast thermography has been poor and contradictory for decades. The heterogeneity of studies and their poor overall quality led national health agencies to conclude that there is not enough scientific evidence to support adding thermography to screening programs. The repeated FDA warnings against the promotion of thermography as a replacement for mammography by alternative medicine clinics, such as spas and homeopathic centers, have had greater weight than the approval of several commercial systems for breast thermography over the years—some of which have since disappeared from the market, largely due to the skepticism of the medical community toward thermography. Medical societies, like the SBI and EUSOBI, are opposed to breast thermography, although the scientific basis for their recommendations tends to be poor and obsolete. We identified several recent studies of relevance for including a large number of patients and/or broader population types, such as asymptomatic women or those with dense breasts. These groups have often been underrepresented in older studies, primarily because the incidence of breast cancer among asymptomatic women is so low that a very large number of patients is required to achieve a sufficient number of positive cases for training and evaluating machine learning methods. Data collection is a time- and resource-consuming process, particularly in the medical field due to the overworked personnel, who often find it challenging to allocate time to collect and deliver the data. Most of these studies were not considered by the aforementioned health societies to base their position against the use of thermography. Some of them were conducted after the publication of the position statements or the systematic reviews commissioned by health authorities—the SBI’s position statement and the paper that supports the EUSOBI’s position are more than a decade old—indicating a potential gap in the evidence base used to inform policy decisions. Even other systematic reviews [73,74,75,76,77], which have a big impact on the opinion of the medical community, have omitted some of these studies.

However, there are recent studies, conducted with reasonable rigor, involving a large number of patients. The meta-analysis by Goñi-Arana et al. [64], which selected those that met strict quality criteria, obtained a pooled sensitivity of 88.5% and a specificity of 71.8%, comparable to those of mammography.

### A Proposal for the Standardization of Breast Thermography Studies

As mentioned in Section 2.2, the meta-analysis in [64] revealed significant variations in sensitivity and specificity values, primarily due to methodological differences among studies in four areas: inclusion criteria, image acquisition, image analysis, and confirmation method. Therefore, acceptance of this technique by radiologists, medical societies, and regulatory agencies requires standardizing these aspects as much as possible. The following suggestions are based on well-known guidelines [78,79], which are more detailed than this section but not specific to breast thermography.

First of all, every study should clearly indicate which subjects are included: asymptomatic women (undergoing either systematic or opportunistic screening), symptomatic patients, or patients suspected of having breast cancer due to previous tests, such as mammography or ultrasound. If the sample contains a mixture of cases, the sensitivity and specificity should be reported for each subpopulation.

Second, the acquisition protocol should be detailed, beginning with patient positioning: in most studies, subjects sit or stand, but in a few of them, they lie prone with both breasts suspended through openings in the top of the imaging bed [47]. In principle, this latter technique allows obtaining more accurate images of the whole breast surface, but the equipment is more expensive, occupies more space (which is often a scarce resource in clinical settings), and is not portable, which is important for rural areas with limited access to hospitals.

Active thermography consists of applying an external stimulus to enhance the contrast between tumors and healthy tissue. One method is the “cold stress challenge,” which involves cooling the breasts with a stream of cold air—as in the 3DIRI system [53]—or cooling another part of the body (for example, by placing the patient’s hands and wrists in ice cold water) and observing how the thermal pattern of the breasts changes over time. In the early 2000s, most breast thermography practitioners abandoned this technique because it is uncomfortable for patients, increases acquisition time, and complicates image interpretation without showing increased accuracy [8]. However, recent research has shown that dynamic thermography is more effective than static image acquisition at identifying malignant tumors [80,81,82], but these are laboratory studies involving very few patients. Other stimuli used for active breast thermography are electric currents, high-frequency electromagnetic fields, magnetic nano-particles, and light [83]. An open research issue is whether the potential increase in diagnostic accuracy outweighs the additional economic costs and patient discomfort.

Studies should also report which infrared cameras were used, specifying their thermal sensitivity and spatial resolution, as these parameters affect the quality of images.

Third, the method used to analyze the thermograms is of utmost importance. In clinics that offer breast thermography as a replacement for mammography, grayscale or color images are usually examined with the naked eye, as was common in the 20th century, which further contributes to the discrediting of this technique. In contrast, all recent studies use computer algorithms that analyze the exact temperature of each pixel. In recent years, deep convolutional networks have become the standard method for medical imaging, including breast thermography [43,84,85,86].

However, most of these algorithms only provide a binary classification. Moreover, sometimes vague classes like *normal* or *abnormal* are used, without explicitly defining them, which makes the studies difficult to interpret and reproduce. Additionally, deep neural networks behave like black boxes, making breast thermography more difficult to accept, because, since the early years of medical AI, it has become clear that physicians are reluctant to accept a machine’s advice if they cannot understand how it was obtained [87]. Explainability is crucial in AI for healthcare for many reasons, including ethical and legal issues. In breast imaging, explanations are essential when different techniques lead to opposing diagnoses and the radiologist must decide which is correct. Therefore, every algorithm for analyzing thermograms should indicate the degree of confidence in its conclusions, preferably in the form of probabilities, as well as the findings these conclusions are based on, such as temperature differences and vascular patterns. For the radiology community to accept breast thermography, it would be highly desirable to have a standard similar to the Breast Imaging Reporting and Data System (BI-RADS), which is currently available for mammography, ultrasound, and MRI.

Finally, studies should indicate which gold standard was used to evaluate the accuracy of thermography, as this is essential to properly evaluate its sensitivity and specificity. For example, in two of the 3DIRI studies [54,55], patients with negative mammography or ultrasound but an abnormal thermogram were referred to MRI. However, due to budgetary constraints, in most studies these cases are counted as false positives of thermography, which results in an underestimation of its specificity. Ideally, all patients should be followed up for at least five years, especially those with suspicious thermograms.

## 5. Conclusions

Breast thermography is innocuous, painless, inexpensive, and portable. Images can be acquired by briefly trained personnel and, when they are analyzed with artificial intelligence, results are immediately available. For these reasons, it can be useful for early cancer detection, particularly among young women, women with dense breasts, and those in low- and middle-income countries. It may also help avoid biopsying benign tumors that appear suspicious under other imaging techniques.

In this paper we have reviewed the origins of breast thermography and the studies that gave this technique a bad reputation. We listed commercial products that were taken off the market despite FDA approval, but we also mentioned a successful system that is now available in 22 countries. Next, we summarized recent studies, some of them with large sample sizes, that yielded high sensitivity and specificity values—in some cases, values similar to or better than those of mammography—especially since the combination of modern infrared cameras with AI algorithms.

We analyzed the position statements of medical societies opposing breast thermography and found that they are based on outdated evidence. Surprisingly, they all cite the BCDDP study, conducted over 50 years ago with low-resolution analog cameras and visual thermogram analysis, without computer support. Sometimes, they also rely on biased assessments of more recent studies. Unfortunately, these recommendations, issued by prestigious scientific societies, could hinder research and progress in breast thermography.

Our analysis suggests that radiologists should be aware of the advances in breast thermography and medical societies should update their position statements in the light of new evidence. They should change their advice against this technique into the recommendation to conduct large-scale, prospective trials, following good practice guidelines, such as those proposed in Section A Proposal for the Standardization of Breast Thermography Studies. Similar to the BI-RADS system established by the American College of Radiology for mammography, ultrasound, and MRI, a standard terminology for reporting thermograms should also be developed. Finally, cost-effectiveness analyses should be conducted. More precisely, the optimal screening pattern for each woman should be determined, incorporating breast thermography in combination with other imaging modalities, based on cost-effectiveness criteria.

## Figures and Tables

**Table 1:** Summary of studies evaluating breast thermography.

	Study	Year	Device	Population	Sensitivity	Specificity
Studies to evaluate commercial systems	Parisky et al. [47]	2003	BCS 2100 (Computerized Thermal Imaging Inc., Ogden, UT, USA)	769 patients scheduled for biopsy	97%	14%
	Arora et al. [50]	2008	Sentinel BreastScan (SBS) (Infrared Sciences Corp., Bohemia, NY, USA)	92 women scheduled for biopsy	96.7%	26.5%
	Wishart et al. [51]	2010	-NoTouch BreastScan (NTBS) (UE Life Sciences Inc., Philadelphia, PA, USA) -SBS	100 patients scheduled for biopsy	70% 48%	48% 74%
	Collet et al. [52]	2014	NTBS	99 patients scheduled for biopsy	78.8%	48.6%
	Sella et al. [53]	2013	3DIRI (Real Imaging Ltd., Airport City, Israel)	256 healthy asymptomatic women and 178 breast cancer patients	90.9%	72.5%
	Sklair-Levy et al. [54]	2016	3DIRI	226 patients at high risk due to genetic predisposition	87.5%	84.32%
	Hellgren et al. [55]	2019	3DIRI	1727 asymptomatic women with dense breasts	58.3%	87%
	Kakileti et al. [56]	2020	Thermalytix (Niramai Health Analytix, Bangalore, India)	470 women -238 symptomatic -232 asymptomatic	91.02% 89.85% 100%	82.39% 69.04% 92.41%
	Singh et al. [57]	2021	Thermalytix	258 symptomatic women	82.5%	80.5%
	Bansal et al. [58]	2023	Thermalytix	459 women (symptomatic and asymptomatic) -168 with dense breasts	95.24% 100%	88.58% 81.65%
Other studies	Gutierrez-Delgado and Vazquez-Luna [59]	2010	DL-700 (Zhejiang Dali Technology Co., Hangzhou, China)	911 women attending cancer screening programs	94.12%	unknown
	Rassiwala et al. [60]	2014	FLIR ThermoVision A-20 (Teledyne Technologies Inc., Thousand Oaks, CA, USA)	1008 asymptomatic women	97.62%	99.17%
	Yao et al. [61]	2014	Wuhan Hao Technology Co. (Wuhan, China)	2036 women with abnormal findings in mammography or ultrasound	84.4%	94.0%
	Garduño-Ramón et al. [62]	2017	FLIR A-300 (Teledyne Technologies Inc., Thousand Oaks, CA, USA)	454 women	86.84%	89.65%
	Wang et al. [63]	2023	InfiRay (IRay Technology Co., Yantai, China)	2202 screening patients	84.51%	83.87%

## Data Availability

No new data were created or analyzed in this study. Data sharing is not applicable to this article.

## Abbreviations

The following abbreviations are used in this manuscript:

MRI	Magnetic resonance imaging
BI-RADS	Breast Imaging Reporting and Data System
BCDDP	Breast Cancer Detection and Demonstration Project
NCI	National Cancer Institute
AI	Artificial intelligence
DMR	Database for Mastology Research
FDA	Food and Drug Administration
SBS	Sentinel BreastScan
NTBS	NoTouch BreastScan
3DIRI	3D Infrared Imaging
SBI	Society of Breast Imaging
EUSOBI	European Society of Breast Imaging
